# Qualidade do Sono Associada ao Nível Habitual de Atividade Física e Sistema Nervoso Autônomo de Fumantes

**DOI:** 10.36660/abc.20190522

**Published:** 2020-12-07

**Authors:** Iara Buriola Trevisan, Luiz Carlos Marques Vanderlei, Mahara Proença, Tiago V. Barreira, Caroline Pereira Santos, Tamara Santos Gouveia, Ercy Mara Cipulo Ramos, Dionei Ramos

**Affiliations:** 1 Universidade Estadual Paulista Júlio de Mesquita Filho Presidente PrudenteSP Brasil Universidade Estadual Paulista Júlio de Mesquita Filho (UNESP),Presidente Prudente, SP - Brasil; 2 Universidade Estadual do Norte do Paraná JacarezinhoPR Brasil Universidade Estadual do Norte do Paraná (UENP), Jacarezinho, PR - Brasil; 3 Syracuse University New York EUA Syracuse University, New York – EUA

**Keywords:** Sono, Qualidade do Sono, Exercício, Frequência Cardíaca, Tabagismo, Doenças do Sistema Nervoso Autônomo

## Abstract

**Fundamento:**

Poucos estudos já examinaram a relação do nível habitual de atividade física e a modulação do sistema nervoso autônomo (SNA) na qualidade do sono de fumantes.

**Objetivos:**

O objetivo deste estudo foi identificar alterações na qualidade do sono de fumantes e sua relação com nível habitual de atividade física e modulação do SNA.

**Métodos:**

Um total de 42 fumantes foram divididos em dois grupos de acordo com o 50º percentil de atividade física de moderada a vigorosa (AFMV). A qualidade do sono foi avaliada utilizando-se o *Mini-Sleep Questionnaire* (mini questionário do sono), e a modulação do SNA foi avaliada por índices de variabilidade de frequência cardíaca (VFC). Para a análise de possíveis diferenças de média, foi utilizada a análise de covariância (ANCOVA) para ajuste de idade, gênero, composição corporal, maços-ano, betabloqueadores, ansiedade, e depressão, em log base 10, exceto por dados qualitativos, tais como gênero e betabloqueadores. Foram estabelecidas correlações utilizando-se a correlação de postos de Spearman. A significância estatística foi definida em 5%.

**Resultados:**

Os fumantes que eram menos ativos demonstraram pior qualidade do sono (p=0,048) e insônia (p=0,045). Além disso, o grupo menos ativo apresentou redução na modulação parassimpática [HF (un; p=0,049); RMSSD (ms; p=0,047) e SD1 (ms; p=0,047)] e aumento do índice de LF (un) index (p=0,033) e razão LF/HF (p=0,040). Houve correlação positiva entre a pontuação total no *Mini-sleep* com o índice de LF (un) (r=0,317, p=0,041) e razão LF/HF (r=0,318, p=0,040) e correlação negativa com o índice de HF (un) (r= -0,322, p=0,038).

**Conclusões:**

Fumantes com baixo nível de atividade física habitual apresentaram baixa qualidade do sono e alterações na modulação do sistema nervoso autônomo. (Arq Bras Cardiol. 2020; [online].ahead print, PP.0-0)

## Introdução

O tabagismo é considerado um grande problema de saúde pública em todo o mundo, apesar de ser uma das causas principais de mortes preveníveis no mundo.^1^ A carga global de doenças crônicas está aumentando e o tabagismo representa um fator de risco importante para o desenvolvimento dessas doenças.^1^

O tabagismo também pode ser responsável por alterações neurocomportamentais, tais como diminuição da memória de trabalho, lapsos de atenção, humor depressivo e distúrbios do sono.^2^ Em relação ao último, vários estudos relatam, em adultos, uma associação negativa entre o tabagismo e a qualidade do sono, tais como insônia,^3^ hipersonia, fragmentação do sono,^4^ sonolência diurna^5^ e má qualidade do sono noturno.^6^

A restrição do sono devido ao tabagismo pode ser causada por vários mecanismos, entre os quais se destaca o impacto da nicotina.^7^ Durante o sono, os níveis de nicotina abaixam, acarretando sintomas de abstinência, que dependem do número de cigarros fumados por dia, nível de dependência da nicotina, e índice de abstinência de nicotina. Além disso, os níveis de monóxido de carbono e a eliminação dos níveis de nicotina no sangue diminuem durante o sono.^7 - 10^

Durante o sono, a modulação do sistema nervoso autônomo (SNA) apresenta mudanças ao longo das transições entre vigília e sono. A modulação parassimpática cardíaca aumenta aproximadamente duas horas antes do início do sono, atinge o pico no início do sono, e diminui no período de sono, enquanto a modulação simpática não muda no início do sono, mas diminui durante estágios de sono mais profundo. Essas alterações produzem a diminuição da frequência cardíaca e o aumento da variabilidade de frequência cardíaca (VFC).^11 , 12^

Fumantes apresentam alterações no SNA caracterizadas por reduções de modulação parassimpática,^13 , 14^ o que sugere que, além de os fumantes apresentarem distúrbios do sono devido ao consumo de cigarros, a redução da modulação parassimpática nesses indivíduos também pode afetar a qualidade do sono.

A literatura sugere que um estilo de vida saudável e ativo pode induzir a um aumento da modulação parassimpática,^15^ promovendo a regulação e o equilíbrio do SNA.^16^ Portanto, um estilo de vida ativo habitual é um dos benefícios à qualidade do sono devido a seus efeitos na regulação do SNA,^17 , 18^ que também pode acontecer com fumantes.^19^ A investigação da relação entre qualidade do sono e modulação do SNA de acordo com o nível habitual de atividade física de fumantes pode gerar informações valiosas para identificar a importância de um estilo de vida mais ativo nesse grupo e, além disso, melhoria da qualidade do sono pode aumentar as chances de índices de sucesso durante a cessação do tabagismo. Portanto, nosso objetivo foi avaliar a qualidade do sono em fumantes e sua relação com o nível habitual de atividade física e a modulação do sistema nervoso autônomo.

## Materiais e Métodos

### Participantes e Procedimentos

Os participantes foram recrutados por anúncios na mídia. Os fumantes foram selecionados independentemente de gênero, com idades entre 18 e 60 anos. Os critérios de inclusão foram: 1) consumir pelo menos 10 cigarros/dia, por pelo menos um ano, 2) ausência de doenças cardiorrespiratórias crônicas pré-existentes que influenciam o SNA significativamente (por exemplo, arritmias, hipertensão não controlada, tosse crônica, bronquite crônica, enfisema pulmonar ou FEV_1_/FVC <70%), 3) não fazer uso excessivo de álcool ou outras drogas ilícitas, 4) não fazer uso de produtos de reposição de nicotina e/ou antidepressivos como auxiliares na cessação do tabagismo. Os critérios de exclusão foram: 1) avaliações incompletas; 2) outliers (mais de 3 desvios padrão de distância da média, indicando erro na coleta de dados de VFC).

**Figura 1:**
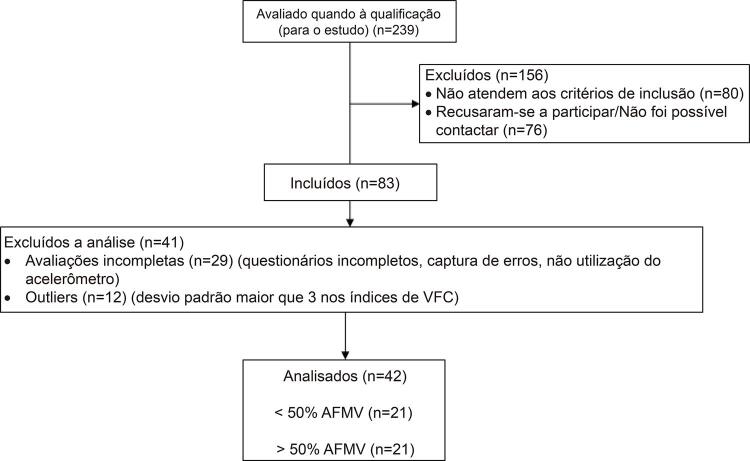
– Fluxograma do estudo. VFC: variabilidade de frequência cardíaca; AFMV: atividade física de moderada a vigorosa.

Um total de 239 fumantes demonstraram interesse em participar do estudo. Dessa forma, 83 participantes foram incluídos, mas 41 participantes foram excluídos devido a avaliações incompletas (n = 29), e participantes que tinham um desvio padrão acima de 3 nos índices de VFC (outliers n = 12). Portanto, 42 participantes foram, então, divididos em dois grupos de acordo com o 50º percentil de atividade física de moderada a vigorosa (AFMV). ( Figura 1 ).

Os participantes foram previamente informados sobre os objetivos da pesquisa e procedimentos e, depois do acordo, assinaram o formulário de consentimento. Este estudo foi aprovado pelo Comitê de Ética da Universidade Estadual Paulista (CAAE: 54550116.6.0000.5402).

As avaliações foram realizadas em dois dias não consecutivos, todos os procedimentos foram realizados no período da manhã em condições controladas de temperatura e umidade relativa do ar (22,0±2,2ºC, 56,6±6,9%), e todos os participantes foram instruídos a não ingerirem álcool, cafeína, analgésicos ou barbitúricos, e a não fazerem exercícios moderados ou vigorosos 13 horas antes da avaliação. Foi realizada a medição de monóxido de carbono exalado (exCO), com um ponto de corte de 10 ppm,^20^ para comprovar a abstinência de nicotina por 24 horas antes das avaliações.^21^ No primeiro dia, os participantes realizaram uma avaliação para coletar dados pessoais, o status do tabagismo, a função pulmonar, dados antropométricos e composição corporal, bem como para fazer uma análise de ansiedade, depressão e qualidade do sono. No segundo dia, todos os participantes foram submetidos à avaliação da modulação do SNA pelos índices de VFC, e do nível habitual de atividade física realizado no acelerômetro. Profissionais previamente treinados acompanharam todas as avaliações.

### Status do Tabagismo

Todos os participantes responderam perguntas sobre o número de cigarros consumidos por dia, a duração do tabagismo, e a dependência da nicotina foi avaliada pelo teste de Fagerström.^22^ O número de maços/anos foi calculado pela fórmula: número de cigarros consumido por dia, dividido por 20 e multiplicado pelo tempo de tabagismo.

### Função Pulmonar

Esse teste foi realizado com um espirômetro portátil (Spirobank 3.6 Medical International Research, Roma, Itália). A interpretação foi feita considerando os padrões da *American Thoracic Society* e da *European Respiratory Society* ,^23^ com valores normais para a população brasileira.^24^

### Composição Corporal

O aparelho octopolar InBody 720 (Copyright®, 1996–2006, da Biospace Corporation, EUA) foi utilizado para calcular o índice de massa corporal (IMC), porcentagem de massa gorda (% MG), massa muscular esquelética (MME) e a massa gorda (MG). O InBody 720 usa oito eletrodos, dois em contato com a palma (E1 e E3) e polegar (E2 e E4) de cada mão, e dois em contato com a ponta (E5 e E7) e o calcanhar (E6 e E8) de cada pé.^25 , 26^

### Ansiedade e Depressão

Estas foram avaliadas pela Escala Hospitalar de Ansiedade e Depressão (HADS - do inglês *Hospital Anxiety and Depression Scale* ).^27^ Este instrumento consiste em uma escala de 14 itens, sete exclusivos para ansiedade e sete exclusivos para depressão.

### Qualidade do Sono

A qualidade do sono foi avaliada pelo *Mini-sleep Questionnaire* ,^28^ validado para a população brasileira por Falavigna et al.,^29^ que consiste em 10 perguntas de autorrelato, com sete respostas possíveis (nunca = 1, muito raramente = 2, raramente = 3, às vezes = 4, frequentemente = 5, muito frequentemente = 6 e sempre = 7). A insônia (perguntas 1, 2, 3 e 7) e hipersonia (perguntas 4, 5, 6, 8, 9 e 10) também são avaliadas por esse instrumento.

### Nível Habitual de Atividade Física

Os participantes foram instruídos a usar um acelerômetro ActiGraph GT3X+ (AG), (ActiGraph LLC, Pensacola, FL, EUA) por um período de 7 dias, inclusive enquanto dormiam, retirando os dispositivos apenas quando estivessem em contato direto com a água (por exemplo, durante o banho ou natação).^30^ O AG foi preso a uma faixa elástica e posicionado no quadril direito. O dispositivo AG é um acelerômetro triaxial e mede a aceleração em 3 planos, gerando contagens de atividades para cada eixo e uma grandeza vetorial representando a combinação de todos os 3 eixos. No presente estudo, os dados brutos foram coletados na frequência de 80 Hz. Dados do dispositivo AG foram baixados utilizando o filtro de baixa extensão do software ActiLife (versão 6.13, ActiGraph LLC), exceto pelos passos/dias em que foram baixados usando o filtro normal. Para a análise de dados, os dados brutos do acelerômetro foram convertidos em contagens e somados em um *epoch* de 60 seg. com a extensão de baixa frequência habilitada.

Um algoritmo previamente validado foi aplicado aos dados do acelerômetro para separar o tempo gasto durante o sono e o tempo gasto acordado.^31 , 32^ Os dados do tempo de uso do sono não foram utilizados na análise dos padrões de atividade descritos abaixo. Períodos de não uso (identificados utilizando-se os dados do acelerômetro AG) foram definidos como blocos consecutivos de pelo menos 60 minutos de contagem zero de atividades, incluindo até 2 minutos consecutivos de contagem de atividades inferiores a 100, de acordo com os critérios do *National Health and Nutrition Examination Survey* (NHANES).^33^ Um dia completo de uso do acelerômetro foi definido como pelo menos 10 horas de uso durante a vigília. Era necessário um mínimo de 4 dias (incluindo pelo menos 1 dia no final de semana) de dados de uso para que o participante pudesse ser incluído na análise de dados.

Após a inspeção e processamento inicial, os dados do tempo de uso foram analisados para determinar quanto tempo os participantes passaram em atividade física de moderada a vigorosa (AFMV), utilizando o ponto de corte definido por Troiano et al. > 2020 *counts* /min (equivalente a 3 MET), intensidade vigorosa (5999 *counts* ou 6 MET).^33^ Cada *epoch* foi classificado como tempo de sedentarismo quando os *counts* do eixo vertical eram <100 *counts* /min.^34^

### Modulação do Sistema Nervoso Autônomo

Para analisar os índices de modulações do SNA, a frequência cardíaca foi captada batimento a batimento, utilizando-se um cardiofrequencímetro (Polar S810i, Finlândia), equipamento previamente validado para captação da frequência cardíaca batimento a batimento e para cálculo dos índices de VFC.^35^

Todos os participantes foram instruídos a não consumir substâncias estimulantes tais como chá, café, refrigerante, chocolate e álcool por 24 horas antes dessa análise. Durante o registro da frequência cardíaca, os participantes foram instruídos a permanecer em silêncio, acordados, em repouso, sem realizar movimentos ou conversar durante a execução, e com respiração espontânea por 20 minutos sentados. Não era permitida a circulação de pessoas na sala durante a execução das coletas, para evitar erros de captura e reduzir a ansiedade dos voluntários.

Os dados obtidos pela monitorização foram transferidos para o computador utilizando o software Polar ProTrainer 5 (versão 5.41.002). Cinco minutos do gráfico são analisados, com pelo menos 256 intervalos RR, selecionados da parte mais estável, depois de filtragem digital, concluída por filtragem manual para eliminar artefatos e batimentos ectópicos. Apenas séries com mais de 95% de batimentos sinusais foram incluídas no estudo.

Os índices de VFC foram calculados nos domínios de tempo e frequência e do *plot* de Poincaré. No domínio do tempo (DT), foram utilizados os índices RMSSD (raiz quadrada média das diferenças sucessivas) e SDNN (desvio padrão de intervalos de normal a normal), ambos expressos em milissegundos (ms). No domínio de frequência, (DF) foram usados componentes de baixa (LF, 0,04 – 0,15 Hz), e alta frequência (HF, 0,15 – 0,40 Hz), em valores absolutos (ms^2^) e em unidades normalizadas (un), bem como a razão LF/HF.^36 , 37^ A análise espectral foi calculada usando o algoritmo da transformada rápida de Fourier.^38^

O *plot* de Poincaré foi utilizado para calcular os seguintes índices: SD1 (desvio padrão da variabilidade instantânea batimento a batimento), SD2 (desvio padrão em longo prazo dos intervalos RR contínuos), e a relação SD1/SD2, que mostra a razão entre as variações curta e longa dos intervalos RR.^39 , 40^ Para a análise dos índices de VFC foi utilizado o software Kubios (Universidade de Kuopio, Finlândia).^41^

### Análise Estatística

Foi realizado um estudo prévio para determinar o tamanho da amostra, e foi estimada uma correlação de r = 0,43 entre qualidade do sono, nível de atividade física e SNA, com erro alfa de 5% e poder amostral de 80%, e uma amostra de 41 participantes foi considerada apropriada.^42^

A amostra foi dividida em dois grupos de acordo com o 50º percentil (26,65 min) de AFMV (<p50 ou >p50). O teste de Shapiro-Wilk analisou a normalidade dos dados. Estatísticas descritivas foram expressas como média e desvio padrão ou média e faixa interquartil (IQR) para variáveis contínuas, e as qualitativas, em frequência (f) e porcentagem (%) para as variáveis categóricas. O teste t não pareado ou o teste Mann-Whitney foram aplicados na comparação entre os percentis nas variáveis de caracterização da amostra. A comparação de qualidade do sono, nível habitual de atividade física e VFC entre percentis foi realizada usando a análise de covariância (ANCOVA) ajustada para idade, gênero, IMC, %MG, MME, maços-ano, betabloqueadores, e depressão, em log base 10 (log10) para diminuir a variabilidade de variáveis não paramétricas, exceto para dados qualitativos, como gênero e betabloqueadores. As premissas para comparar duas amostras independentes foram testadas, examinando a normalidade dos dados, homogeneidade entre os grupos, conforme o teste de Levene e relação linear entre as covariáveis e as variáveis dependentes. As correlações entre qualidade do sono, VFC e nível habitual de atividade física foram calculadas utilizando-se a correlação de postos de Spearman, já que os dados eram não paramétricos de acordo com o teste de Shapiro-Wilk. Todas as análises foram realizadas utilizando o software SPSS (versão 22.0) e a significância estatística foi estabelecida em 5%.

## Resultados

A Tabela 1 apresenta informações sobre as características gerais da população estudada. O grupo de fumantes menos ativos (<p50 AFMV) tinha mais mulheres (81%) do que homens (19%) comparada com o grupo mais ativo (>p50 AFMV). A % de MG era mais alta no grupo <p50 AFMV (p=0,017), enquanto a MME era mais alta no grupo >p50 AFMV (p=0,015).

**Tabela 1:** – Características gerais dos fumantes dos 50° percentis de AFMV (<p50 ou >p50)

Características demográficas	<p50 (N=21)	>p50 (N=21)	p valor
Gênero (F/M)	17/4	8/13	0,005†*
Idade (anos), média (DP)	42,0 (10,8)	44,3 (8,9)	0,644§
**Composição Corporal**			
Altura (cm), média (DP)	1,6 (0,1)	1,7 (0,1)	0,138§
Peso (kg), média (DP)	70,1 (12,6)	74,6 (15,1)	0,302§
IMC (kg/m^2^), média (DP)	26,6 (4,5)	26,5 (4,2)	0,893§
IMC (kg/m^2^), média (DP)	34,4 (6,6)	29,0 (7,6)	0,017§*
MME (kg), média (IQR)	23,3 (22,2–27,2)	29,5 (24,2–34,7)	0,015‡*
MG (kg), média (DP)	24,5 (7,6)	22,0 (8,5)	0,323§
**Status do tabagismo**			
Tempo de tabagismo (anos), média (DP)	25,3 (11,5)	26,5 (9,2)	0,724§
Cigarros por dia, média (IQR)	20,0 (12,0–20,0)	20,0 (10,0–30,0)	0.827‡
Cigarros por dia, média (IQR)	22,0 (13,5–31,9)	24,8 (13,3–35,0)	0,537‡
Dependência de nicotina, média (DP)	5,2 (2,3)	5,6 (2,3)	0,594§
**HADS**			
Ansiedade, média (DP)	7,4 (4,5)	9,3 (3,8)	0,144§
Depressão, média (DP)	6,1 (4,0)	6,1 (2,7)	1§
**Índices espirométricos**			
FVC (% pred), média (DP)	94,1 (12,4)	94,4 (19,4)	0,968§
FEV1 (% pred), média (DP)	93,5 (12,1)	91,1 (19,1)	0,629§
FEV1/FVC (% pred), média (DP)	99,0 (6,0)	96,2 (5,5)	0,120§
PFE (% pred), média (IQR))	76,0 (72,0–87,0)	76,5 (58,8-90,3)	0,657‡
FEV1/FVC (% pred), média (DP)	94,7 (31,8)	86,3 (26,5)	0,365§
**Medicamentos atuais, f (%)**			
Cardiovascular	6 (29)	4 (19)	0.469†
Betabloqueadores	1 (17)	1 (25)	
Bloqueadores AT1	4 (67)	3 (75)	
Inibidores da ECA	1 (17)	0 (0)	
Antidepressivos	7 (33)	3 (14)	0,147†
Metabólicos	1 (5)	1 (5)	1†

Dados expressos como média e desvio padrão ou média e faixa interquartil (IQR) e frequência (f) e porcentagem (%). F/M: Feminino/Masculino; IMC: índice de massa corporal; MME: massa muscular esquelética; MG: massa gorda; FVC: capacidade vital forçada; FEV1: volume expiratório forçado no primeiro segundo; FEV1/FVC: razão de FEV1 e FVC; PEF: fluxo expiratório de pico; FEF25-75%: fluxo expiratório entre 25% e 75% de FVC. * p valor para diferença estatística significativa; †Teste qui-quadrado; § Teste t não pareado; ‡ teste Mann-Whitney. Fonte: preparado pelo autor.

A Tabela 2 mostra as variáveis de qualidade do sono, nível habitual de atividade física e VFC de fumantes nos percentis de <p50 e <p50 de AFMV, ajustadas para fatores confusos tais como idade, gênero, IMC, %MG, MME, betabloqueadores, maços-ano, ansiedade e depressão. Observamos que os fumantes menos ativos (<p50) apresentaram má qualidade do sono de acordo com as pontuações totais no Mini-sleep, insônia e menos passos/dias de AFMV, comparada àqueles com nível mais alto de AFMV (>p50). Para os índices de VFC, o grupo menos ativo (<p50) demonstrou redução da modulação parassimpática expressa por RMSSD, HF (un) e índices de SD1, e aumento de LF (un) e razão LF/HF, quando comparado com o grupo mais ativo (>p50).

**Tabela 2:** – Qualidade do sono, nível de atividade física e modulação autonômica cardíaca dos fumantes dos 50° percentis de AFMV (<p50 ou >p50)

Mini-sleep	< p50 (N=21)	< p50 (N=21)	p†
Total, média (IQR)	34,0 (28,5–38,5)	29,0 (22,5–32,5)	0,048*
Insônia, média (IQR)	14,0 (8,0–19,0)	10,0 (7,0–14,0)	0,045*
Hipersonia, média (IQR)	20,0 (16,5–22,5)	17,0 (13,0–22,0)	0,113
**Nível de atividade física**			
AFMV (min), média (IQR))	14,0 (7,4–19,1)	38,0 (30,4–48,6)	<0,0001*
Sedentário (min), média (DP)	450,5 (147,0)	466,4 (100,3)	0,939
Passos/Dia, média (IQR)	7058,0 (5874,5–8431,0)	9753,0 (7977,5–11354,5)	0,020*
**VFC**			
RR médio (ms), média (DP)	751,8 (71,2)	805,3 (96,6)	0,161
SDNN (ms), média (DP)	32,2 (12,7)	33,2 (14,4)	0,982
FC média (bpm), média (DP))	80,7 (7,9)	75,6 (9,0)	0,147
RMSSD (ms), média (IQR)	14,6 (10,1–26,4)	18,8 (14,6–31,5)	0,047*
Índice triangular RR, média (DP)	8,7 (3,1)	9,1 (3,6)	0,970
TINN (ms), média (DP)	142,9 (57,8)	138,8 (66,6)	0,648
LF (ms^2^), média (IQR))	220,0 (91,5–607,0)	264,0 (71,5–526,0)	0,530
HF (ms^2^), média (IQR))	101,0 (23,5–206,0)	114,0 (47.5–269,5)	0,351
LF (un), média (IQR))	74,5 (57,3–82,3)	70,4 (54,0 -79,0)	0,033*
HF (un), média (IQR))	25,5 (17,5–42,6)	28,7 (21,0–45,9)	0,049*
LF/HF (ms^2^), média (IQR)	2,9 (1,4–4,8)	2,5 (1,2–3,8)	0,040*
SD1 (ms), média (IQR)	10,3 (7,2–18,7)	13,3 (10,3–22,3)	0,047*
SD2 (ms), média (IQR)	43,5 (17,0)	43,6 (18,8)	0,670
SD1/SD2 (ms^2^), média (IQR)	0,3 (0,3–0,4)	0,3 (0,3–0,4)	0,457

Dados expressos como média e desvio padrão ou média e faixa interquartil (IQR). AFMV: atividade física de moderada a vigorosa; nu: unidades normalizadas; RR: entre batimentos cardíacos sucessivos; SDDN: Desvio padrão de intervalo de normal a normal; FC: frequência cardíaca; RMSSD: Raiz quadrada média de diferenças sucessivas; TINN: interpolação triangular de intervalos de RR; LF: baixa frequência; HF: alta frequência; SD1: desvio padrão da variabilidade batimento a batimento instantânea; SD2: desvio padrão dos intervalos de RR contínuos de longo prazo. * p valor para diferença estatística significativa; †ANCOVA ajustada para idade, gênero, IMC, %MG, MME, maços-ano, betabloqueadores, ansiedade e depressão. Fonte: preparado pelo autor.

**Figura 2:**
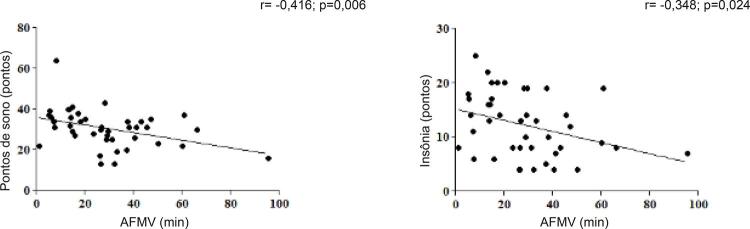
– Análise de correlação entre qualidade do sono e nível habitual de atividade física. AFMV: atividade física de moderada a vigorosa; r: Postos de Spearman; p: significância estatística (0,05).

A Figura 2 mostra que houve uma correlação negativa moderada entre AFMV (min) e pontuação total no Mini-sleep e insônia.

**Figura 3:**
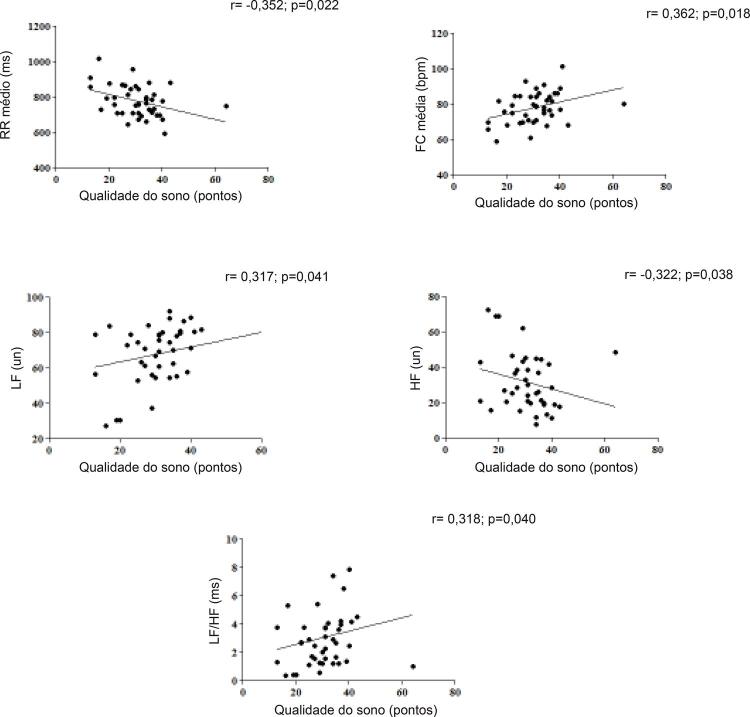
– Análise de correlação entre qualidade do sono VFC. RR: entre batimentos cardíacos sucessivos; FC: frequência cardíaca; LF: frequência baixa; HF: frequência alta; r: Postos de Spearman; p: significância estatística (0,05).

A Figura 3 mostra que houve uma correlação positiva de fraca a moderada entre a pontuação total no Mini-sleep o índice de LF (un) e a razão LF/HF, e uma correlação negativa de fraca a moderada com o RR médio e o índice de HF (un).

## Discussão

O estudo teve o objetivo de avaliar a qualidade do sono em fumantes e sua relação com o nível habitual de atividade física e a modulação do SNA. Portanto, os resultados mostraram que fumantes com níveis mais baixos de atividade física habitual tinham má qualidade do sono e insônia, bem como diminuição da modulação parassimpática e aumento de índice de LF (un) e razão LF/HF.

Os fumantes têm mais chance de desenvolver distúrbios do sono do que os não fumantes.^4 , 10 , 42^

A literatura indica que a nicotina é um dos principais mecanismos responsáveis pelos distúrbios no sono em tabagistas, devido aos efeitos independentes e interativos de seus neurotransmissores nos mecanismos centrais que regulam o ciclo sono-vigília, aumentando a latência do sono.^10 , 43 , 44^ De acordo com McNamara et al.,^44^ para cada cigarro consumido, há uma diminuição de 1,2 minutos no tempo total de sono, o que sugere uma possível influência da nicotina como causa potencial dessa relação dose-resposta. Além disso, a diminuição nos níveis de nicotina durante o sono produz sintomas relacionados à síndrome de abstinência, que aumenta a insônia nessa população.^7^

Também podem ocorrer distúrbios do sono nesses indivíduos devido à presença de doenças pulmonares que podem surgir por causa do tabagismo (por exemplo, câncer de pulmão e obstrução pulmonar crônica)^45^ e variáveis comportamentais, ou seja, quando um indivíduo usa o cigarro como forma de aliviar tensões, probabilidade de baixa qualidade de vida, e o surgimento de sintomas de depressão e ansiedade.^2 , 3 , 46^

Considerando a forte evidência sobre a relação entre tabagismo e qualidade do sono, alguns estudo investigaram a influência da atividade física para melhorar a qualidade do sono.^19 , 47^ Segundo Chen et al.,^19^ fumantes inativos (0-999 kcal/semana) têm um índice mais alto de ocorrência de insônia em comparação com os fumantes ativos (≥1000 kcal/semana) conforme as atividades de lazer e não lazer. Masood et al.,^47^ observaram que fumantes pesados tinham mais probabilidade de ter menos de cinco horas de sono por dia e de ter comportamentos não saudáveis, tais como sedentarismo, dieta ruim e consumo de álcool. Assim como esses estudos, nossos resultados também mostraram que fumantes com nível de atividade física de moderada a vigorosa abaixo de 26,65 min/dia também exibiram má qualidade do sono e insônia. Entretanto, ainda é necessário investigar os vários níveis de atividade física nessa doença.

Uma das hipóteses para melhorar atividade física qualidade do sono com prática regular de atividade física é a adaptação psicológica, como melhoria do humor, diminuição da secreção de cortisol, aumento do consumo de energia e fadiga, que aumenta a necessidade de dormir para recuperar as energias, além das mudanças na composição corporal.^18 , 48^ Em relação a este último ponto, nossos resultados demonstraram que os fumantes fisicamente mais ativos com boa qualidade do sono apresentam % MG mais baixa e MME mais alta.

Além disso, a prática da atividade física, especialmente a realizada continuamente, é capaz de acarretar mudanças em FC e VFC.^49^ Em indivíduos treinados, ocorrem aumentos na modulação parassimpática, que podem estar relacionados à melhoria do humor, qualidade do sono, tempo de latência e uso de medicamentos para melhorar a qualidade do sono de adultos e dos idosos.^17 , 49 , 50^

Indivíduos com insônia apresentam aumento de FC durante o sono, diminuição do tempo de sono total, e diminuição dos índices de VFC, o que pode dificultar transições dos estágios do sono, o que exige a atividade parassimpática para chegar a estágios mais profundos.^51^ Em fumantes, essas mudanças podem ser mais evidentes, porque o tabagismo pode levar à redução de VFC.^13 , 14 , 52^ Bodin et al.,^52^ avaliaram fumantes em períodos durante os quais eles consumiram e não consumiram cigarros por 12 horas, e observaram que, depois de fumar, os participantes apresentaram redução de VFC, com uma redução de HF e intervalos de RR quando comparados a períodos em que não fumaram. Em fumantes pesados, Santos et al.,^14^ observaram aumento de índice de LF (un) e de LF/HF, e a diminuição do índice HF (un) e da razão SD1/SD2, em comparação com fumantes moderados.

Entretanto, nossos resultados demonstraram que o nível de atividade física em fumantes estava associado a VFC, mesmo que seja uma população com alterações em VFC devido ao tabagismo. Os fumantes mais ativos fisicamente aumentaram a modulação parassimpática expressa por índices de RMSSD, HF(un) e SD1, e diminuição do índice LF(un) e razão LF/HF, comparado com fumantes menos ativos, o que sugere que a prática de atividade física nessa população melhora as condições, e essas evidências podem, pelo menos parcialmente, estar relacionadas a mudanças no SNA.

Na análise da correlação entre qualidade do sono e índices de VFC, observa-se que a má qualidade do sono estava associada a níveis mais altos da frequência cardíaca, índice LF (un) e razão LF/HF, e a níveis mais baixos de modulação parassimpática, sugerindo que a baixa qualidade do sono e a insônia podem estar correlacionadas à redução da VFC, especialmente em fumantes menos ativos.

Podemos destacar, como limitações do estudo, a falta de um grupo de controle de não fumantes para avaliar melhor a influência do tabagismo nos aspectos estudados, a não determinação da fase de ciclo menstrual de mulheres na pré-menopausa, e uso de medicamentos antidepressivos, que podem influenciar o SNA. Esses itens podem ser realizados em estudos futuros. Além disso, os índices de VFC são influenciados por idade, gênero, medicamentos cardiovasculares, e isso pode ter afetado os resultados. Entretanto, as análises foram ajustadas quanto a possíveis fatores confusos.

## Conclusão

Em resumo, este estudo demonstrou que a qualidade do sono de fumantes e sua relação com nível habitual de atividade física e modulação do SNA. Portanto, além da nicotina, a pior qualidade do sono pode estar associada a níveis mais baixos de atividade física e alterações na modulação do sistema nervoso autônomo, sugerindo que a promoção de atividade física em fumantes pode ajudar a melhorar a qualidade do sono e garantir maior controle autônomo. Entretanto, são necessários novos estudos que avaliem níveis diferentes de atividade física na modulação do SNA durante o sono em comparação com indivíduos saudáveis, o que pode evitar distúrbios do sono, incentivar um estilo de vida saudável à medida que incentiva a cessação do tabagismo.
