# Supplementary material for: Qualidade do Sono Associada ao Nível Habitual de Atividade Física e Sistema Nervoso Autônomo de Fumantes
Source: Arq Bras Cardiol. 2020 Dec 7;116(1):26–35. [Article in Portuguese] doi: 10.36660/abc.20190522 (PMC8159490; doi:10.36660/abc.20190522)
Supplement: Translation

# Translation: Sleep Quality Associated with Habitual Physical Activity Level and Autonomic Nervous System of Smokers

**Authors:** Iara Buriola Trevisan, Luiz Carlos Marques Vanderlei, Mahara Proença, Tiago V. Barreira, Caroline Pereira Santos, Tamara Santos Gouveia, Ercy Mara Cipulo Ramos, Dionei Ramos

**Affiliations:** 1 Brazil Universidade Estadual Paulista Júlio de Mesquita Filho (UNESP), Presidente Prudente, SP - Brazil; 2 Brazil Universidade Estadual do Norte do Paraná (UENP), Jacarezinho, PR - Brazil; 3 EUA Syracuse University, New York – EUA

**Keywords:** Sleep, Sleep,Quality, Exercise. Heart Rate, Tobacco Use Disorder, Autonomic Nervous System Diseases

## Abstract

**Background:**

Few studies have examined the relationship of one’s habitual physical activity level and autonomic nervous system (ANS) modulation on sleep quality in smokers.

**Objective:**

The aim of this study was to identify changes in the sleep quality of smokers and its relation with their habitual physical activity level and ANS modulation.

**Methods:**

Forty-two smokers were divided into two groups according to the 50th percentile of the moderate-to-vigorous physical activity (MVPA). Sleep quality was assessed using the Mini-sleep Questionnaire, and ANS modulation was assessed by indices of heart rate variability (HRV). To examine the possible mean differences, the analysis of covariance (ANCOVA) was used, adjusted for age, sex, body composition, pack-years, beta-blockers, anxiety, and depression in log base 10, not including qualitative data, such as sex and beta-blockers. Correlations were made by using the Spearman rank correlation. The statistical significance was set at 5%

**Results:**

The smokers who were less active showed poor sleep quality (p=0.048) and insomnia (p=0.045). Furthermore, the less active group presented decreased parasympathetic modulation [HF (un; p=0.049); RMSSD (ms; p=0.047) and SD1 (ms; p=0.047)] and an increased LF (un) index (p=0.033) and LF/HF ratio (p=0.040). A positive correlation between the total Mini-sleep score with LF (un) index (r=0.317, p=0.041) and LF/HF ratio (r=0.318, p=0.040) and negative correlation with HF (un) index (r= -0.322, p=0.038).

**Conclusions:**

Smokers with lower levels of habitual physical activity showed poor sleep quality and alterations in autonomic nervous system modulation; (Arq Bras Cardiol. 2020; [online].ahead print, PP.0-0)

## Introduction

Smoking is considered a major public health problem worldwide, despite being a major cause of preventable death worldwide.^1^ The global burden of chronic diseases is increasing and smoking represents an important risk factor for the development of these diseases.^1^

Smoking may also be responsible for neurobehavioral alterations, such as reduced working memory, lapses of attention, depressed mood, and sleep disturbances.^2^ In the latter respect, several studies conducted with adults reported a negative association between smoking and sleep quality, such as insomnia,^3^ hypersomnia, sleep fragmentation,^4^ daytime sleepiness,^5^ and poor nocturnal sleep quality.^6^

Sleep restriction due to smoking can be caused by several mechanisms, the most prevalent of which is the impact of nicotine.^7^ During sleep, the nicotine levels decrease, triggering withdrawal symptoms, which are dependent on the number of cigarettes smoked per day, the nicotine dependence level, and the rate of nicotine withdrawal. Moreover, carbon monoxide levels and the elimination of nicotine levels in the blood decrease during sleep.^7 - 10^

During sleep, the autonomic nervous system (ANS) modulation presents changes along transitions between wakefulness and sleep. The cardiac parasympathetic modulation increases approximately two hours before sleep onset, reaches the peak at sleep onset, and decreases in the sleep period, while sympathetic modulation does not change at sleep onset but does decrease during the deeper stages of sleep. These changes produce decreased heart rate and increased heart rate variability (HRV).^11 , 12^

Smokers show changes in ANS characterized by reductions in parasympathetic modulation,^13 , 14^ suggesting that in addition to smokers presenting sleep disturbances due to cigarette consumption, the decrease in parasympathetic modulation in these individuals may also affect one’s sleep quality.

The literature suggests that a healthy and active lifestyle is able to induce an increase in parasympathetic modulation,^15^ promoting ANS regulation and balance.^16^ Thus, a habitual active lifestyle, is one of the benefits to sleep quality due to its effects on ANS regulation,^17 , 18^ which can also happen with smokers.^19^ Investigating the relationship between sleep quality and ANS modulation according to the habitual physical activity level of smokers can promote valuable information to identify the importance of a more active lifestyle in this population. Moreover, improvement in sleep quality may increase the chances of success rates during smoking cessation. Therefore, the present study aimed to assess the sleep quality in smokers and its relationship with the habitual physical activity level and ANS modulation.

## Materials and Methods

### Participants and Procedures

Participants were recruited through announcements in the media. Smokers, 18 to 60 years of age and regardless of sex, were selected. The inclusion criteria were: 1) consume at least 10 cigarettes/day, for at least one year; 2) absence of known pre-existing chronic cardiorespiratory diseases that significantly influence the ANS (e.g arrhythmias, uncontrolled hypertension, chronic cough, chronic bronchitis, pulmonary emphysema, or FEV_1_/FVC <70%); 3) No excessive use of alcohol or other illicit drugs; 4) No use of nicotine replacement products and/or antidepressants as an aid to stop smoking. The exclusion criteria were: 1) incomplete assessments and 2) Outliers (more than 3 standard deviations away from mean, indicating error in collected HRV data).

A total of 239 smokers expressed interest in participating in the study. Thus, 83 participants were included, but 41 participants were excluded due to incomplete assessments (n = 29) and participants who had a standard deviation greater than 3 in the HRV indices (outliers, n = 12). Therefore, 42 participants were then divided into two groups according to the 50^th^percentile of the moderate-to-vigorous physical activity (MVPA) ( Figure 1 ).

**Figure 1:** – Flow chart of the study. HRV: heart rate variability, MVPA: moderate-to-vigorous physical activity.

Participants were previously informed about the research objectives and procedures and, after agreement, signed the consent form. This study was approved by the Research Ethics Committee of Sao Paulo State University (CAAE: 54550116.6.0000.5402).

The assessment was performed on two non-consecutive days; all procedures were performed in the morning under controlled temperature and relative humidity (22.0±2.2ºC, 56.6±6.9%), and all participants were instructed not to ingest alcohol, caffeine, anesthetics, or barbiturates, nor to perform moderate or vigorous exercise 24 hours prior to assessment. Measurement of exhaled carbon monoxide (exCO), with a cut-off point of 10 ppm,^20^ was performed to prove nicotine abstinence from 24 hours prior to the assessments.^21^ On the first day, the participants took part in an assessment to collect personal data, as well as data regarding smoking status, pulmonary function, and anthropometric and body composition, along with an analysis of anxiety, depression, and sleep quality. On the second day, all participants underwent an assessment of ANS modulation by HRV indices and of the habitual physical activity level performed by the accelerometer. Previously trained professionals accompanied all the assessments.

### Smoking Status

All participants answered questions from the Fagerström questionnaire concerning the number of cigarettes consumed per day, time of smoking, and nicotine dependence.^22^ The pack/years was calculated by the formula: number of cigarettes consumed per day divided by 20 and multiplied by the time of smoking.

### Pulmonary Function

The pulmonary function was analyzed using a portable spirometer (Spirobank 3.6 Medical International Research, Rome, Italy). The interpretation was made considering the standards of American Thoracic Society and European Respiratory Society,^23^ with normal values recorded for the Brazilian population.^24^

### Body Composition

The InBody 720 octopolar apparatus (Copyright®, 1996–2006, by Biospace Corporation, USA) was used to calculate the body mass index (BMI), percent fat mass (%FM), skeletal muscle mass (SMM), and fat mass (FM). The InBody 720 uses eight electrodes, two in contact with the palm (E1 and E3) and thumb (E2 and E4) of each hand and two in contact with the front (E5 e E7) and heel (E6 and E8) of each foot.^25 , 26^

### Anxiety and Depression

Anxiety and depression were assessed by applying the Hospital Anxiety and Depression Scale (HADS) questionnaire.^27^ This instrument consists of a 14-item scale, seven exclusive for anxiety and seven exclusive for depression.

### Sleep Quality

The sleep quality was assessed by a Mini-sleep Questionnaire,^28^ validated for the Brazilian population by Falavigna et al.,^29^ which consists of 10 self-reported questions with seven possible answers (never = 1, very rarely = 2, rarely = 3, sometimes = 4, often = 5, very often = 6, and always = 7). Insomnia (questions 1, 2, 3, and 7) and hypersomnia (questions 4, 5, 6, 8, 9, and 10) are also assessed in this instrument.

### Habitual Physical Activity Level

Participants were instructed to wear an ActiGraph GT3X+ (AG) accelerometer, (ActiGraph LLC, Pensacola, FL USA) for a 7-d period, including while sleeping, only removing the devices when coming in direct contact with water (i.e. bathing or swimming).^30^ The AG was attached to an elastic waistband and positioned on the right hip. The AG device is a triaxial accelerometer and measures acceleration in 3 planes, generating activity counts for each axis and a vector magnitude representing the combination of all 3 axes. In the current study, the raw data was collected at a frequency of 80 Hz. Data from the AG device was downloaded using the low extension filter from the ActiLife software (version 6.13, ActiGraph LLC), not including the steps/day, which was downloaded using the normal filter. For data analysis, raw accelerometer data was converted into counts and summed over a 60 sec period with the low frequency extension enabled.

A previously validated algorithm was applied to the AG accelerometer data to separate sleep wear time from awake wear time.^31 , 32^ Data from sleep wear time was not used in the analysis of the activity patterns described below. Periods of non-wear (identified using the AG accelerometer data) were defined as consecutive blocks of at least 60 min of zero activity counts, including up to 2 consecutive minutes of activity counts less than 100, in line with the National Health and Nutrition Examination Survey (NHANES) criteria.^33^ A complete day of acclerometer use was defined as at least 10 hours of wake wear time. A minimum of 4 days (including at least 1 weekend day) of wear data was necessary in order for participants to be included in the data analysis.

After initial inspection and processing, accelerometer data from awake wear time was analyzed to determine how much time participants spent in moderate-to-vigorous physical activity (MVPA) using the cut point by Troiano et al. > 2020 counts/min (equivalent to 3 METs), vigorous intensity (5,999 counts or 6 METs).^33^ Each period was classified as sedentary time if vertical axis counts were < 100 counts/min.^34^

### Autonomic Nervous System (ANS) Modulation

To analyze the indices of ANS modulations, the heart rate was captured beat by beat, using a cardio-frequency (Polar S810i, Finland) equipment, which had been previously validated for the capitation of one’s heart rate, beat by beat, and its use for calculating HRV indices.^35^

All participants were instructed not to consume stimulating substances, such as tea, coffee, soda, chocolate, and alcohol for 24 hours prior to this analysis. While recording the heart rate, participants were instructed to remain silent, awake, at rest, without performing movements and conversations during execution, and with spontaneous breathing for 20 minutes while sitting. The circulation of people was not allowed in the room during the execution of data collection in order to avoid capture errors and reduce the anxiety of volunteers.

Data obtained by monitoring were transferred to the computer using the software Polar ProTrainer 5 (version 5.41.002) and each five minutes of the chart were analyzed, with at least 256 RR intervals, selected from the most stable part of the chart, after digital filtration, completed via manual filtering to eliminate artifacts and ectopic beats; only series with over 95% of sinus beats were included in the study.

HRV indices were calculated in the time and frequency domains and the Poincaré plot. In the time domain (TD), the indices of Root Mean Square of Successive Differences (RMSSD) and Standard Deviation of Normal to Normal intervals (SDNN) were calculated, both expressed in milliseconds (ms). In the frequency domain (FD), this study used the low frequency spectrum component (LF, 0.04 – 0.15 Hz) and the high frequency (HF, 0.15 – 0.40 Hz), in absolute values (ms^2^) and in normalized units (un), as well as the LF/HF ratio.^36 , 37^ The spectral analysis was calculated using the fast Fourier transform algorithm.^38^

The Poincaré plot was used to calculate the following indices: SD1 (standard deviation of the instantaneous beat to beat variability); SD2 (standard deviation of the long-term continuous R-R intervals); and the SD1/SD2 ratio, which shows the ratio between short and long-term variations of the RR intervals.^39 , 40^ To analyze the HRV index, the Kubios software (University of Kuopio, Finland) was used.^41^

### Data Analysis

Previous research was used to determine the sample size. A correlation of r = 0.43 between sleep quality, physical activity level, and ANS was estimated, with an alpha error of 5% and a sample power of 80%. Hence, a sample of 41 participants was deemed appropriate.^42^

The sample was divided into two groups according to the 50^th^percentile (26.65 min) of the MVPA (<p50 or >p50). The Shapiro-Wilk test analyzed data normality. The continuous variables were described as mean and standard deviation or as median and interquartile range (IQR), except for categorical variables which were described as frequency (f) and percentage (%). Unpaired t test or Mann-Whitney test were applied in the comparison between the percentiles in the characterization variables of the sample. Comparison of sleep quality, habitual physical activity level, and HRV between percentiles was performed using covariance analysis (ANCOVA) adjusted for age, sex, BMI, %FM, SMM, pack-years, beta-blockers, anxiety, and depression in log base 10 (log10) to decrease the variability of nonparametric variables, not including qualitative data, such as sex and beta-blockers. The assumptions for comparing two independent samples were tested by examining the normality of the data, homogeneity between the groups, according to the Levene’s test, and the linear relationship between the covariates and the dependent variables. Correlations between sleep quality, HRV, and habitual physical activity level were calculated using Spearman’s rank correlation, which was used because, according to the Shapiro-Wilk test, the data proved to be nonparametric. All analyzes were performed using software SPSS (version 22.0) and statistical significance was set at 5%.

## Results

Table 1 presents information about the general characteristics of the studied population. The group of less active smokers (<p50 MVPA) had more women (81%) than men (19%), as compared to the more active group (>p50 MVPA). The % FM was higher in the <p50 MVPA (p=0.017), whereas, the SMM was higher in the >p50 MVPA group (p=0.015).

**Table 1:** – General characteristics of smokers in 50th percentiles of MVPA (<p50 or >p50)

Demographic characteristics	<p50 (N=21)	>p50 (N=21)	p value
Sex (F/M)	17/4	8/13	0.005†*
Age (years), mean (SD)	42.0 (10.8)	44.3 (8.9)	0.644§
**Body Composition**			
Height (cm), mean (SD)	1.6 (0.1)	1.7 (0.1)	0.138§
Weigh (kg), mean (SD)	70.1 (12.6)	74.6 (15.1)	0.302§
BMI (kg/m^2^), mean (SD)	26.6 (4.5)	26.5 (4.2)	0.893§
%FM, mean (SD)	34.4 (6.6)	29.0 (7.6)	0.017§*
SMM (kg), median (IQR)	23.3 (22.2–27.2)	29.5 (24.2–34.7)	0.015‡*
FM (kg), mean (SD)	24.5 (7.6)	22.0 (8.5)	0.323§
**Smoking status**			
Smoking duration (years), mean (SD)	25.3 (11.5)	26.5 (9.2)	0.724§
Cigarettes days, median (IQR)	20.0 (12.0–20.0)	20.0 (10.0–30.0)	0.827‡
Pack-years, median (IQR)	22.0 (13.5–31.9)	24.8 (13.3–35.0)	0.537‡
Nicotine dependence, mean (SD)	5.2 (2.3)	5.6 (2.3)	0.594§
**HADS**			
Anxiety, mean (SD)	7.4 (4.5)	9.3 (3.8)	0.144§
Depression, mean (SD)	6.1 (4.0)	6.1 (2.7)	1§
**Spirometric indices**			
FVC (% pred), mean (SD)	94.1 (12.4)	94.4 (19.4)	0.968§
FEV1 (% pred), mean (SD)	93.5 (12.1)	91.1 (19.1)	0.629§
FEV1/FVC (% pred), mean (SD)	99.0 (6.0)	96.2 (5.5)	0.120§
PFE (% pred), median (IQR)	76.0 (72.0–87.0)	76.5 (58.8-90.3)	0.657‡
FEF25-75% (% pred), mean (SD)	94.7 (31.8)	86.3 (26.5)	0.365§
**Current medications, f (%)**			
Cardiovascular	6 (29)	4 (19)	0.469†
Beta-blockers	1 (17)	1 (25)	
AT1-blockers	4 (67)	3 (75)	
ACE inhibitors	1 (17)	0 (0)	
Antidepressant	7 (33)	3 (14)	0.147†
Metabolic	1 (5)	1 (5)	1†

Data expressed as mean and std. deviation or median and interquartile range (IQR) and frequency (f) and percentage (%). F/M: Female/Male; BMI: body mass index; SMM: skeletal muscle mass; FM: fat mass; FVC: forced vital capacity; FEV1: forced expiratory volume in the first second; FEV1/FVC: ratio of FEV1 and FVC; PEF: peak expiratory flow; FEF25-75%: expiratory flow between 25% and 75% of FVC. * p-value for significant statistical difference; †Chi-square test; § Unpaired t test; ‡ Mann-Whitney test. Source: author himself

Table 2 shows the variables of sleep quality, habitual physical activity level, and HRV of smokers in the <p50 and >p50 percentiles of MVPA, which was adjusted for confounding factors, such as age, sex, BMI, %FM, SMM, beta-blockers, pack-years, anxiety, and depression. It was observed that less active smokers (<p50), as compared to those with a higher MVPA level (>p50), showed poor sleep quality according to the total scores regarding Mini-sleep, insomnia, lower MVPA, and steps/days. As regards the HRV indices, the less active group (<p50) showed a decreased parasympathetic modulation, expressed by the RMSSD, HF(un), and SD1 indices, and an increased LF(un) and LF/HF ratio when compared to the more active group (>p50).

**Table 2:** – Sleep quality, physical activity level, and autonomic cardiac modulation of smokers in 50th percentiles of MVPA (<p50 or >p50)

Mini-sleep	< p50 (N=21)	> p50 (N=21)	p†
Total, median (IQR)	34.0 (28.5–38.5)	29.0 (22.5–32.5)	0.048*
Insomnia, median (IQR)	14.0 (8.0–19.0)	10.0 (7.0–14.0)	0.045*
Hypersomnia, median (IQR)	20.0 (16.5–22.5)	17.0 (13.0–22.0)	0.113
**Physical activity level**			
MVPA (min), median (IQR)	14.0 (7.4–19.1)	38.0 (30.4–48.6)	<0.0001*
Sedentary (min), mean (SD)	450.5 (147.0)	466.4 (100.3)	0.939
Steps/Day. median (IQR)	7058.0 (5874.5–8431.0)	9753.0 (7977.5–11354.5)	0.020*
**HRV**			
Mean RR (ms), mean (SD)	751.8 (71.2)	805.3 (96.6)	0.161
SDNN (ms), mean (SD)	32.2 (12.7)	33.2 (14.4)	0.982
Mean HR (bpm), mean (SD)	80.7 (7.9)	75.6 (9.0)	0.147
RMSSD (ms), median (IQR)	14.6 (10.1–26.4)	18.8 (14.6–31.5)	0.047*
RR triangular index, mean (SD)	8.7 (3.1)	9.1 (3.6)	0.970
TINN (ms), mean (SD)	142.9 (57.8)	138.8 (66.6)	0.648
LF (ms^2^), median (IQR)	220.0 (91,5–607.0)	264.0 (71.5–526.0)	0.530
HF (ms^2^), median (IQR)	101.0 (23,5–206.0)	114.0 (47.5–269.5)	0.351
LF (nu), median (IQR)	74.5 (57.3–82.3)	70.4 (54.0 –79.0)	0.033*
HF (nu), median (IQR)	25.5 (17.5–42.6)	28.7 (21.0–45.9)	0.049*
LF/HF (ms^2^), median (IQR)	2.9 (1.4–4.8)	2.5 (1.2–3.8)	0.040*
SD1 (ms), median (IQR)	10.3 (7.2–18.7)	13.3 (10.3–22.3)	0.047*
SD2 (ms), mean (SD)	43.5 (17.0)	43.6 (18.8)	0.670
SD1/SD2 (ms^2^), median (IQR)	0.3 (0.3–0.4)	0.3 (0.3–0.4)	0.457

Data expressed as mean and std. deviation or median and interquartile range (IQR). MVPA: moderate to vigorous physical activity; nu: normalized units; RR: between successive heart beats; SDNN: Standard Deviation of Normal to Normal interval; HR: heart rate; RMSSD: Root Mean Square of Successive Differences; TINN: triangular interpolation of RR intervals; LF: low frequency; HF: high frequency; SD1: standard deviation of the instantaneous beat to beat variability; SD2: standard deviation of the long-term continuous R-R intervals. * p-value for significant statistical difference; †ANCOVA adjusted for age, sex, BMI, %FM, SMM, pack-years, Beta-blockers, anxiety, and depression. Source: author himself.

Figure 2 shows that there was a moderate negative correlation between MVPA (min) and total Mini-sleep score and insomnia.

**Figure 2:** – Correlation analysis between sleep quality and habitual physical activity level. MVPA: moderate-to-vigorous physical activity; r: Spearman’s rank; p: statistical significance (0.05).

Figure 3 shows that there was a weak to moderate positive correlation between the total Mini-sleep score with Mean HR (l/min), LF (un) index, and LF/HF ratio, as well as a weak to moderate negative correlation with Mean RR (ms) and HF (un) index.

**Figure 3:** – Correlation analysis between sleep quality and HRV. RR: between successive heart beats; HR: heart rate; LF: low frequency; HF: high frequency; r: Spearman’s rank; p: statistical significance (0.05).

## Discussion

This study aimed to assess smokers sleep quality and its relationship to one’s habitual physical activity level and ANS modulation. Therefore, our results showed that smokers with a lower level of habitual physical activity had poor sleep quality and insomnia, as well as a decrease in the parasympathetic modulation and an increase in the LF (un) index and LF/HF ratio.

Smokers are more likely to develop sleep disturbances than nonsmokers.^4 , 10 , 42^

The literature indicates that nicotine is one of the main mechanisms responsible for sleep disturbances in smokers, due to the independent and interactive effects of their neurotransmitters on the central mechanisms that regulate the sleep-wake cycle, increasing sleep latency.^10 , 43 , 44^ According to McNamara et al.,^44^ for each cigarette consumed, there is a decrease of 1.2 min in total sleep time, which suggests a possible influence of nicotine as a potential cause of this dose-response relationship. Furthermore, the decrease in nicotine levels during sleep produces symptoms related to the withdrawal syndrome, which increases insomnia in this population.^7^

Sleep disturbances in these individuals may also occur due to the presence of pulmonary diseases that may arise due to smoking (e.g, lung cancer and chronic obstructive pulmonary disease)^45^ and behavioral variables, i.e., when the individual uses cigarettes as stress relief, because of a likelihood of a poor quality of life, and due to the appearance of depression and anxiety symptoms.^2 , 3 , 46^

Given the strong evidence about smoking on poor sleep quality, some studies have investigated the influence of physical activity on improved sleep quality.^19 , 47^ According to Chen et al.,^19^ inactive smokers (0-999 kcal/week) have a higher rate of insomnia when compared to active smokers (≥1000 kcal/week), when considering leisure and non-leisure activities. Masood et al.,^47^ observed that heavy smokers were more likely to have less than five hours of sleep per day and more likely to take on unhealthy behaviors, such as a sedentary lifestyle, poor diet, and alcohol consumption. In addition to these studies, our results showed that smokers with moderate to vigorous physical activity levels below 26.65 min/day presented poor sleep quality and insomnia. However, there is still a need to investigate the different levels of physical activity in this condition.

One of the hypotheses to improve sleep quality through the regular practice of physical activity involves physiological adaptations, such as mood improvement, decreasing cortisol secretion, increase in energy consumption, and fatigue that increases the need to sleep for energy restoration, besides changes in body composition.^18 , 48^ Regarding this last point, our results showed that more physically active smokers with good sleep quality present lower %FM and higher SMM.

Furthermore, the practice of physical activity, especially that performed continuously, is capable of causing changes in HR and HRV.^49^ In trained individuals, increased parasympathetic modulation occurs, which may be related to one’s improvement in mood, sleep quality, latency time, and use of medications to improve sleep quality in both adults and the elderly.^17 , 49 , 50^

Individuals with insomnia present increased HR during sleep, decreased total sleep time, and decreased HRV indices, which may hinder transitions of the stages of sleep, in turn requiring parasympathetic activity to achieve deeper stages.^51^ In smokers, these changes may be more evident, because smoking may lead to a reduction in HRV.^13 , 14 , 52^ Bodin et al.^52^ evaluated smokers in periods in which they consumed and did not consume cigarettes for 12 hours and observed that after smoking the participants presented a reduction in HRV, with a decrease in HF and RR intervals when compared to non-smoking periods. In heavy smokers, Santos et al.,^14^ observed increased LF(un) and LF/HF indices and a decreased HF(un) index and SD1/SD2 ratio when compared to moderate smokers.

However, our results demonstrated that the physical activity level in smokers was associated with HRV even though it is a population with changes in HRV due to smoking. More physically active smokers presented increased parasympathetic modulation, expressed by the RMSSD, HF (un), and SD1 indices, as well as a decrease in the LF (un) index and LF/HF ratio when compared to less active smokers. This finding suggests that the practice of physical activity in this population improves sleep conditions; such evidence may, at least partly, be related to changes in the ANS.

In the analysis of correlation between sleep quality and HRV indices, it was observed that poorer sleep quality was associated with higher levels of heart rate, LF(un) index, and LF/HF ratio, as well as lower levels of parasympathetic modulation, suggesting that poor sleep quality and insomnia may be correlated with a reduction in HRV, especially in less active smokers.

Limitations of this study include the lack of a control group of non-smokers to better evaluate the influence of smoking on the studied aspects, the non-determination of the phase of menstrual cycles of women in premenopause, and antidepressant medication, which may influence the ANS. Future studies on these issues are warranted. Furthermore, HRV indices are influenced by age, sex, and cardiovascular medication, which may have influenced the results. However, the analyzes were adjusted for potential confounding factors.

## Conclusion

In summary this study showed that the sleep quality of smokers was associated with one’s physical activity level and ANS modulation. Thus, in addition to nicotine, the poorer sleep quality may be associated with a lower level of physical activity and alterations in autonomic nervous system modulation, suggesting that promoting physical activity in smokers may help improve sleep quality and better autonomic control. However, there is a need for new studies that evaluate different levels of physical activity in ANS modulation during sleep as compared to healthy individuals, which may prevent sleep disorders and encourage a healthy lifestyle by encouraging patients to stop smoking.
